# Editorial: Coaches' Role in Youth Sports Performance: Early Specialization Versus Long-Term Development

**DOI:** 10.3389/fpsyg.2021.774944

**Published:** 2021-10-26

**Authors:** Jorge E. Morais, Daniel A. Marinho, Flávio A. De Souza Castro, Tiago M. Barbosa

**Affiliations:** ^1^Department of Sport Sciences, Polytechnic Institute of Bragança (IPB), Bragança, Portugal; ^2^Research Center in Sports Health and Human Development (CIDESD), University of Beira Interior, Covilhã, Portugal; ^3^Department of Sport Sciences, University of Beira Interior, Covilhã, Portugal; ^4^Escola de Educação Física, Grupo de Pesquisa em Esportes Aquáticos, Universidade Federal do Rio Grande do Sul, Porto Alegre, Brazil

**Keywords:** coach, athlete, relationship, development, talent identification

Youth sports are planned sports programs for children and adolescents with designated coaches, organized practices, and scheduled competitions. Such programs can be organized and implemented at schools (by physical education teachers instead of coaches), as well as in other sports organizations (i.e., federations, associations, local clubs). Primary aim should be to focus on providing young athletes with fundamental motor skills in tandem to their maturation stage. Indeed, these programs are aimed at mass participation rather than on developing elite athletes. The participation in such programs during childhood and adolescence showed to have major benefits in children's and adolescent's physical, psychological, and social development. On the other hand, youth sports programs can also serve as a link to talent identification and development programs aiming to identify young athletes with potential for success in adult/elite sport. As they are mass orientated, many youth athletes can be observed which will increase the likelihood of talent identification. Afterwards, these athletes can be guided to high-performance programs aiming to achieve eventually an elite level.

This Research Topic aimed to gather new evidence about: (1) youth sports performance and its determinant factors; (2) share insights on different development programs in youth sports (e.g., short-term vs. long-term development) within the same sport and between sports; (3) better understand the effect of coaches' guidance on young athletes (performance, psychological, biological and others). Five articles were included in this Research Topic after going under peer-review. Based on these articles it seems that the long-term athlete development (LTAD), coach-athlete relationship, long-term impact of youth sport coaching, youth sports development pathways are main concerns. Not surprisingly, due to the pandemic period that washed the world since 2021, the challenges faced to reorganize coach training programs are included. One study aimed to analyse the association between coaches' experience and their perceptions on the implementation of a LTAD model for swimmers (Costa et al.). The authors presented a new way to assess how the sports policies implemented by the National Sports Federations reach end-users in the field, such as coaches. It was enhanced that coaches are given a substantial role in a process that, along with the National Swimming Federation, can enhance youth engagement throughout their sports careers and increase the chance to reach an elite level. Moreover, it was suggested that a national tracking system can be developed with the aim to match “certification levels” × “athletes group” and to monitor if every coach has the minimum certification level to be in charge of a specific group at the beginning of the season. Others (Nash and Taylor) aimed to examine the long-term impact of coaching tennis by using observations, field notes and interviews as data sources. The longitudinal nature of this research highlighted the need for coach developers and sporting organizations to reconsider the biopsychosocial significance, talent development, engagement with sport (in study case: tennis), and the implications for coaching practice. The authors encouraged the necessity for coherence and criticality in the complex milieu of youth sport coaching contexts and the development of expertise in coaching.

The study by van der Berg et al. aimed to investigate youth sports development pathways through both models of development within a South African context. The student-athlete group within the South African university context has diverse development pathways as they transition from secondary to tertiary education. The differentiation in skills and competitive participation levels of athletes as they enter university, create practical complications for coaches to manage the talent environment. Coaches may find it difficult to support the varied developmental levels of students as they transition into university sport and this is evident from the low satisfaction of student-athletes in terms of inadequate supportive and challenging environment, fundamental development aspects, support networks and long-term development focus. In another specific context, Li aimed to understand the relevant research on coach–athlete relationship theory, moral leadership, and team effectiveness theory in the Chinese context. All this considering how to maximize team performance. This study proved that the coach–athlete relationship has a positive predictive relationship with moral leadership and verifies the chain mediation effect of moral leadership and trust between coach–athlete relationship and team effectiveness. The study by Santos et al. aimed to provide insights about the challenges that stakeholders in Portugal and across the globe may face throughout the Coronavirus Disease 2019 pandemic to reorganize coach training programs and suggest strategies to help coaches learn. The authors acknowledge the need to develop changes to coach training programs in Portugal and across the world during the Covid-19 pandemic but suggest that decision-makers along with other stakeholders reflect on these changes and make informed decisions that consider existent knowledge on coach training and learning. In a time of crisis, coach training programs face many challenges that need to be addressed and can also help us reflect on how to change our understandings of learning and increase our effectiveness as teachers and coach developers.

Overall, based on these five studies, it must be enhanced the key-role that coaches have in a skill acquisition process. Themselves, and the support that must be given to them by the sports system, are fundamental for the athletes' development as humans and sportsmen. Moreover, it should be highlighted that a phenomenon like the COVID-19 pandemic, immediately put in risk the sports system especially the coach-athlete relationship.

When children are involved in sports, their physical, psychological, and social development is enhanced. Besides learning and developing a variety of useful skills, they also grow into adulthood with an authentic set of characteristics and values. At the same time, they are also increasing the likelihood of talent identification. In this sense, studies about this topic should be encouraged to understand how children behave while practicing sports, the balance between benefits and disadvantages, understand the talent identification programs, and if the sports system/organizations support athletes and coaches in a proper way.

## Author Contributions

All authors listed have made a substantial, direct and intellectual contribution to the work, and approved it for publication.

## Conflict of Interest

The authors declare that the research was conducted in the absence of any commercial or financial relationships that could be construed as a potential conflict of interest.

## Publisher's Note

All claims expressed in this article are solely those of the authors and do not necessarily represent those of their affiliated organizations, or those of the publisher, the editors and the reviewers. Any product that may be evaluated in this article, or claim that may be made by its manufacturer, is not guaranteed or endorsed by the publisher.

